# Mitogenomes of Polar Bodies and Corresponding Oocytes

**DOI:** 10.1371/journal.pone.0102182

**Published:** 2014-07-17

**Authors:** Luca Gianoarli, Donata Luiselli, Anna Maria Crivello, Martin Lang, Anna Pia Ferraretti, Sara De Fanti, M. Cristina Magli, Giovanni Romeo

**Affiliations:** 1 SISMER Reproductive Medicine Unit, Bologna, Italy; 2 Alma Mater Studiorum, University of Bologna, Bologna, Italy; 3 European School of Genetic Medicine, Bologna, Italy; Institute of Zoology, Chinese Academy of Sciences, China

## Abstract

The objective of the present study was to develop an approach that could assess the chromosomal status and the mitochondrial DNA (mtDNA) content of oocytes and their corresponding polar bodies (PBs) with the goal of obtaining a comparative picture of the segregation process both for nuclear and mtDNA. After Whole Genome Amplification (WGA), sequencing of the whole mitochondrial genome was attempted to analyze the segregation of mutant and wild-type mtDNA during human meiosis. Three triads, composed of oocyte and corresponding PBs, were analyzed and their chromosome status was successfully assessed. The complete mitochondrial genome (mitogenome) was almost entirely sequenced in the oocytes (95.99% compared to 98.43% in blood), while the percentage of sequences obtained in the corresponding PB1 and PB2 was lower (69.70% and 69.04% respectively). The comparison with the mtDNA sequence in blood revealed no changes in the D-loop region for any of the cells of each triad. In the coding region of blood mtDNA and oocyte mtDNA sequences showed full correspondence, whereas all PBs had at least one change with respect to the blood-oocyte pairs. In all, 9 changes were found, either in PB1 or PB2: 4 in *MT-ND5*, 2 in *MT-RNR2*, and 1 each in *MT-ATP8*, *MT-ND4*, *MT-CYTB*. The full concordance between oocyte and blood in the 3 triads, and the relegation of changes to PBs, revealed the unexpected coexistence of different variants, giving a refined estimation of mitochondrial heteroplasmy. Should these findings be confirmed by additional data, an active mechanism could be postulated in the oocyte to preserve a condition of ‘normality’.

## Introduction

As mitochondria are maternally inherited, the pool carried by the mature oocyte is the final complement for the conceptus throughout his life. In the oocyte, mitochondria provide the amount of ATP needed to undergo the fertilization process and in the embryo the energy necessary to acquire developmental competence. It is only after implantation that mitochondria start to replicate. In the previous stages, the total amount of mitochondria in each daughter cell becomes reduced at each cell division: a few of them will be removed from the ooplasm by segregation into polar bodies (PBs), while in the preimplantation embryo, the number of mitochondria will be approximately halved at each mitosis. This situation implies that any defect with phenotypic consequences will probably have adverse effects on oocyte and embryo development respectively, both at preimplantation and postimplantation stages [1].

Several lines of evidence indicate that dysfunctions in mitochondrial bioenergetic activities represent a major cause of chromosomal nondisjunction during meiotic and mitotic divisions, as well as of the reduced viability of oocytes and embryos. These defects seem to be especially relevant in advanced reproductive age as the damage by oxidative stress is more pronounced with negative effects on further development [2].

The mitochondrial complement of the mature oocyte originates from a progenitor pool of 10–20 mitochondria in the primordial germ cells that have experienced repeated replication cycles during the growth phase of oogenesis [3]. One of the peculiarities of these cytoplasmic organelles is represented by the presence of their own genome (mtDNA) that encodes for 13 proteins, which are subunits of the respiratory complexes. Because of its lack of protective histones, low capacity to repair DNA damage and proximity to the respiratory chain (a potent source of mutagenic free radicals), mtDNA is characterized by a very high sequence mutation rate, on the order of 10–20 times that of comparable nuclear DNA genes [4].

Considering that all genes encoded by mtDNA are critical for life, many of these frequently occurring mutations could be deleterious and result in a genetic load extremely harmful for the species. Nevertheless, a selection system in the ovary was postulated that is able to prevent those oocytes carrying severe mtDNA mutations to be ovulated and fertilized [5], [6]. In this way, new deleterious mutations have extremely low chances to contribute to the genetic load of the species. In other words, the massive destruction by atresia of millions and millions of human oocytes during the female reproductive lifetime can then be seen not as inefficiency, but rather as a strategy permitting selection against severe mtDNA mutations, while maintaining a high mtDNA sequence evolution rate [4].

In this perspective, *de novo* mtDNA mutations, some of which might potentially alter mitochondrial energy metabolism, are constantly introduced into the female germline, but their expression rarely occurs. It must be considered that they represent a source of bioenergetic diversity with a potential role in providing adaptation to different environmental conditions. Females harbouring a condition of heteroplasmy, that is a mixture of mutant and wild-type mtDNA, can therefore transmit a varying proportion of mutant mtDNA to their offspring.

The mechanism regulating mtDNA segregation during oogenesis is still completely unknown, and a mitochondrial genetic bottleneck has been postulated to occur at the end of gametogenesis in an individual and mutation specific manner [7]. A recent study reported that the mutant-load differences between the first polar body (PB1) and its corresponding oocyte was higher in highly mutated PBs, suggesting that a selection process could act against highly mutated cells during gametogenesis [8]. Our work compared the levels of 3 pathogenic mtDNA mutations in 51 first polar bodies (PBs) and their counterpart (oocytes, blastomeres, or whole embryos), by semiquantitative fluorescent PCR and restriction endonuclease digestion. As no information was available for second polar bodies (PB2), the modality of mtDNA segregation at female meiosis could not be entirely represented. Therefore, the existence of a mechanism trying to relegate abnormalities to PBs to preserve the corresponding oocyte could not be fully supported. This hypothesis was actually formulated for nuclear chromosome segregation following the observation of reciprocal aneuploidy events resulting in the formation of euploid oocytes [9].

In order to obtain a comprehensive picture of the segregation for both nuclear and mtDNA, we designed a method capable to assess the chromosomal status and to detect mtDNA sequence variants in oocytes and corresponding PBs.

## Materials and Methods

The standardization of methods implied the use of enough quantity of mtDNA from PBs and oocytes for Sanger sequencing. The technique of Whole Genome Amplification (WGA) was chosen because previous studies had shown that, even when applied to degraded or limited amounts of DNA, WGA has a specificity and reproducibility close to 100% in detecting point mutations [10], [11].

The DNA from 3 triads made of donated oocytes (generated by 3 different women) and their corresponding PBs was extracted and amplified by WGA using random primer extension with non-self-complementary primers (PicoPlex WGA, Rubicon Genomics, MI, USA). An aliquot of the amplified product was used to detect aneuploidy by array-CGH (Comparative Genomic Hybridization) [12], while a second aliquot (diluted 1∶10) was used to amplify the whole mitochondrial genome (coding and non-coding regions) using specific primers in a final volume of 10 µL (MitoALL Resequencing kit, Applera, Foster City, CA).

The PCR products were purified by ExoSAP-IT and sequenced with BigDye kit version 3.1 on a ABI 3730 Genetic Analyzer automated sequencing machine (Applera, Foster City, CA). Electropherograms were inspected and aligned to the mitochondrial genome reference sequence (NC_012920) with SeqScape version 2.5 software (Applera, Foster City, CA).

Sequences obtained from blood (B) oocytes (O) PB1 and PB2 are deposited in GenBank (accession numbers: KJ937460–KJ937471). The nucleotide changes detected upon comparison within each triad were analyzed by four different predictors (Polyphen-2, SNP&GO, Mutation Assessor, and MutPred) to calculate the possible impact of a mtDNA base change on the structure and function of the resulting polypeptide chain.

The study was approved by the competent Institutional Review Board (IRB; Comitato Etico indipendente AUSL di Bologna, Prot. N 752/CE, 12 July 2011). All patients signed the specific informed consent that was approved by the IRB.

## Results and Discussion

**Figure 1:**
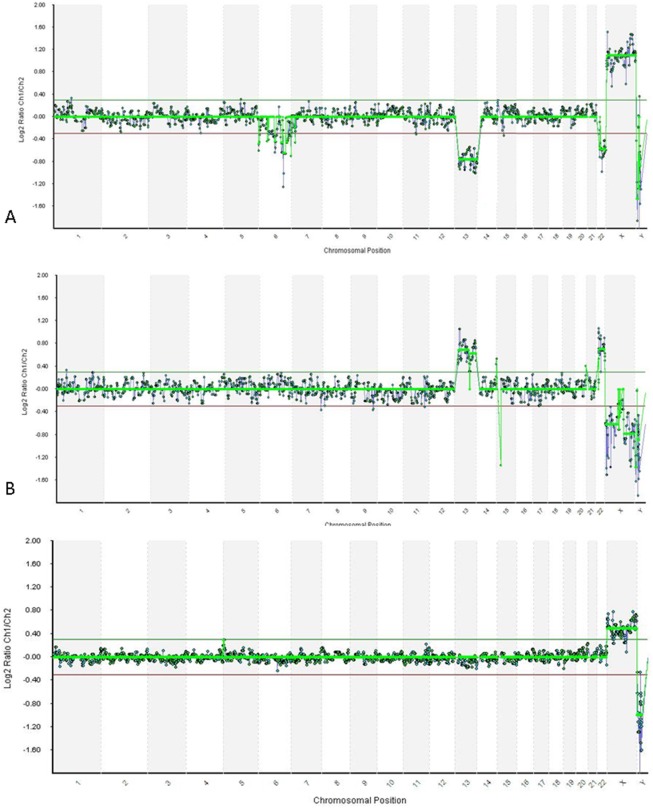
Results from array-Comparative Genomic Hybridization in one triad. (A) The first polar body shows a gain for chromosome X and a loss for chromosomes 13 and 22. (B) The second polar body shows a gain for chromosomes 13 and 22, and a loss for chromosome X. The anomalies for the three chromosomes are reciprocal as confirmed by the euploid condition of the corresponding oocyte.

The chromosome status of the 3 oocytes and corresponding PBs was successfully assessed. Oocyte 1 was euploid following reciprocal aneuploidies for chromosomes X, 13 and 22 in the corresponding PBs (Fig. 1). Oocyte 2 and 3 were both aneuploid due to the presence of multiple abnormalities in both PBs of oocyte 2, and of a single aneuploidy in PB2 of oocyte 3.

**Figure 2:**
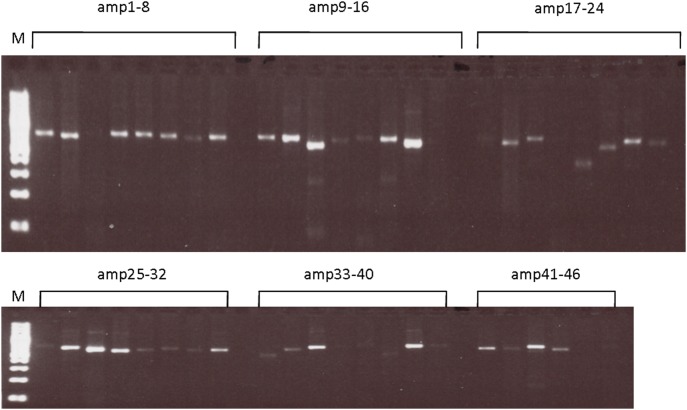
Amplification of the entire mitochondrial genome from polar body obtained by the amplification of 46 overlapping fragments using MitoAll kit.

The complete mitochondrial genome (16569 bp) was almost entirely sequenced in blood DNAs as well as in the oocytes (98.43% and 95.99% respectively), while the percentage of sequences obtained in the corresponding PB1 and PB2 was lower (69.70% and 69.04% respectively) (Figure 2). The sequence analysis of the D-loop region revealed no changes between blood, the oocytes and the corresponding PBs.

For the remaining coding region, sequences for the pairs of blood mtDNA and oocyte mtDNA showed full correspondence, whereas all PBs showed at least one change with respect to the blood-oocyte pairs. As reported in table 1, 9 changes were found: 4 in *MT-ND5*, 2 in *MT-RNR2* and 1 each in *MT-ATP8, MT-ND4, MT-CYTB* (Full information reported in Table S1).

**Table 1:** Distribution of the 9 detected mutations in the 3 studied triads.

		Blood	Oocyte	PB1	PB2
Triad 1	*MT-ND5*	wt	wt	wt	12684A
Triad 2	*MT-ATP8*	wt	wt	8446G	Nd
	*MT-ND4*	wt	wt	wt	11736C
	*MT-CYTB*	wt	wt	15543T	wt
Triad 3	*MT-RNR2*	wt	wt	2232insA	Nd
	*MT-RNR2*	wt	wt	2392C	Nd
	*MT-ND5*	12612G	12612G	wt	12612G
	*MT-ND5*	wt	wt	12684A	wt
	*MT-ND5*	12705C	12705C	wt	12705C

wt: wild type.

Nd: not detected.

Regarding the distribution of the mutations, only one change was detected in the first triad and was found in PB2. In the second triad there were 3 changes, 2 in PB1 and one in PB2. Finally, 5 changes were detected in the third triad, all being in PB1. In two cases, the changes appeared as back-mutations. In all cases presented in this study the observed mutations are compatible with the phylogenetically established mutations (both in the coding region and in the D-loop) which characterize the haplogroups as identified in the peripheral blood of the donor woman. As an example, mutation 12684A which appears in *ND5* in the first triad is perfectly compatible with haplogroup H3, as defined by two different predictors (Hmtdb, http://www.hmtdb.uniba.it/; and Haplofind, https://haplofind.unibo.it/). In the same manner, the mutations in triads 2 and 3 are compatible with haplogroups J1c1 and J1c3 of donor women 2 and 3 respectively. On the basis of these observations, it seems highly unlikely that in all cases WGA introduced by chance new mutations compatible with each haplogroup.

To estimate the possible impact of the detected mtDNA base changes on the structure and function of the resulting polypeptide chain, four different predictors (see Methods) were used. While seven of the observed changes were silent, the remaining two, mapping in the *MT-ND4* and *MT-CYTB* regions, were predicted to have a high and a medium functional impact, respectively. However, the corresponding oocytes were wild-type at the locus.

The limited number of cells analyzed in this study does not permit to draw final conclusions on the biological significance of the observed results. However, the similar behaviour of the 3 triads showing full concordance between oocyte and blood, while relegating potential *de novo* and harmful mtDNA mutations to PBs is certainly intriguing. These data demand the extension of the approach we used to more cases in order to obtain a complete picture of the mtDNA segregation during meiosis in oocytes.

There are several possible explanations for why mutations are more frequently seen in PBs than in oocytes or blood.

The most likely explanation reflects the recent evidence that very low-level heteroplasmic variants are present in all healthy individuals and are likely to be due to both inherited and somatic single base substitutions which in oocytes and blood could remain under the detection threshold of Sanger sequencing [13]. This would imply that pre-existing mtDNA variants would appear at random in PB 1 and 2 due to the bottleneck effect caused by the reduction in number of mitochondria occurring in PBs. Accordingly, since PB1 and PB2 are the products of two different bottlenecks events, it is not surprising that the changes presented in table 1 are always discordant. An even more interesting hypothesis can be put forward based on recent evidence on the selective propagation of functional mtDNA during oogenesis in *Drosophila,* which restricts the transmission of a deleterious mitochondrial variant [14]. This in turn is in keeping with the strong purifying selection demonstrated in the mouse female germline [5], [6].

Evidence for selective processes in inheritance of mutant mtDNA and accumulation of mitochondrial heteroplasmy across the human genome with advanced age in peripheral blood has also been provided [15]. All these data reveal the unexpectedly dynamic nature of human heteroplasmy, suggesting that both *de novo* mutations and selective pressure affect blood mtDNA sequences over the course of human lifespan. Since all these findings indicate a previously uncharacterized mechanism for the selection of wild-type mtDNA, which may be evolutionarily conserved to limit the transmission of deleterious mutations, it will be interesting to confirm by Next Generation Sequencing (NGS) the presence of low levels of heteroplasmic mutant mtDNA in oocytes and to follow its segregation in PBs. It could then be hypothesized that the oocyte has an active mechanism to preserve a condition of ‘normality’, similar to what has been observed for chromosome segregation during meiosis [8], [9]. This postulated mechanism could guide the extrusion of mtDNA variants in the PBs to prevent the transmission of severe mutations causing an altered mitochondrial energy metabolism. At the same time, the controlled accumulation of mtDNA variants in other cell lineages might produce a bioenergetic diversity that might become advantageous in new environments.

Finally the data reported here show that WGA of the DNA extracted from PBs and oocytes allows the analysis of mitochondrial sequences after specific amplification of mtDNA. This strategy is powerful to the point that even very low copy numbers of variant mtDNA can be detected in PBs making it possible to reconstruct the segregation pattern of mitogenomes during meiosis in a manner analogous to what has been achieved for nuclear genomes using amplified DNA from single oocytes and PBs [16].

## Supporting Information

Table S1 Complete list of the detected mitochondrial DNA nucleotide changes in polar bodies and corresponding oocyte in the 3 studied triads. Results from blood sequencing are also reported.
(XLSX)
