# Correction: Mitogenomes of Polar Bodies and Corresponding Oocytes

**DOI:** 10.1371/journal.pone.0112000

**Published:** 2014-10-22

**Authors:** The PLOS ONE Staff

The first author's name is spelled incorrectly. The correct name is Dr. Luca Gianaroli, and the correct citation is:

Gianaroli L, Luiselli D, Crivello AM, Lang M, Ferraretti AP, et al. (2014) Mitogenomes of Polar Bodies and Corresponding Oocytes. PLoS ONE 9(7): e102182. doi:10.1371/journal.pone.0102182
